# London Times

**Published:** 1855-05

**Authors:** 


London Items.—Dr. Watson is not now connected with any hospital, but is in the enjoyment of a fine practice in the "West End." He never visits patients in the "city." In stature, he is quite imposing. Upon an immense pair of shoulders reposes an immense head, with a prodigious anterior development. Added to these imposing features is an air of the greatest gravity. His measured and deep-toned words issue from his larynx like the low, solemn notes of some huge church organ. He seems almost incapable of excitement.

Sir Benjamin Brodie is the antipodes, complete of Dr. Watson. I had labored under the silly but not very singular notion that Sir Benjamin was a man of six feet one or two inches, with massive shoulders, and everything else in keeping, whereas in fact he is about five feet six inches, and weighs not more than 120 lbs. While, however, his stature or "bodily presence is contemptible," his eye has something of genius in it. His face is rather aquiline, and exhibits much activity and vivacity of expression. He speaks freely and fluently, and seems to have paid some attention to politics as well as to surgery. From the activity of mind and body which he possesses, it is to be hoped he will yet long remain an ornament to the profession. His practice is chiefly, I believe, of a consulting character.

Feeling some interest in the subject of *Porter*, finding it often proper to use it myself in the summer, and frequently prescribing it for others, and moreover having heard such disgusting accounts of the mode of brewing in London, I concluded to visit one of these establishments. I was very politely shown over the entire establishment of Messrs. Read & Co., and was gratified to find that there is no truth whatever in the common opinion, (so common that it has gotten into medical books of high authority,) that the London porter is made from the foulest water of the filthy docks of that great city. One of the agents informed me that many years ago, before the Thames was the common sewer of the city, that it is probable those breweries near the river procured the necessary amount of water from thence, but that such was not the case now. The water at Messrs. Read & Co's. establishment is procured by means of two wells between 200 and 300 feet deep, passing through a thick stratum of chalk, in order to avoid the city drainage. From these wells the water is thrown by steam to the top of the immense establishment, whose tanks receive it, and from whence it is distributed to the "cooling rooms," &c. This water is pure and delightful.—*Stethoscope.*

